# Melatonin alleviates vascular endothelial cell damage by regulating an autophagy‐apoptosis axis in Kawasaki disease

**DOI:** 10.1111/cpr.13251

**Published:** 2022-05-17

**Authors:** Yuanzheng Zheng, Saihua Huang, Jialing Zhang, Jia Hou, Fang Wu, Wenji Wang, Xiao Han, Yonghao Gui

**Affiliations:** ^1^ Cardiovascular Center Children's Hospital of Fudan University Shanghai China; ^2^ National Health Commission (NHC) Key Laboratory of Neonatal Diseases Fudan University Shanghai China; ^3^ Institute of Pediatrics, Children's Hospital of Fudan University, National Children's Medical Center Fudan University Shanghai China; ^4^ Department of Clinical Immunology Children's Hospital of Fudan University Shanghai China; ^5^ Department of Neonatology, Shanghai General Hospital Shanghai Jiao Tong University School of Medicine Shanghai China

## Abstract

**Objectives:**

Melatonin has been reported to be an appropriate candidate for mitigating various cardiovascular injuries, owing to its versatility. This study aimed to explore the role of melatonin in Kawasaki disease (KD)‐associated vasculitis and its underlying mechanisms.

**Material and Methods:**

The role of melatonin was evaluated in human coronary artery endothelial cells (HCAECs), peripheral blood mononuclear cells from KD patients, human THP1 cell line in vitro, and a *Candida albicans* water‐soluble fraction (CAWS)‐induced KD mouse model in vivo. Cell proliferation assay, cell apoptosis assay, cell co‐culture, RNA extraction, RNA sequencing, reverse transcription quantitative PCR, enzyme‐linked immunosorbent assay (ELISA), transwell assay, western blot, dual‐luciferase reporter assay, and autophagic flux assay were performed to investigate the function and regulatory mechanisms of melatonin in vitro, while haematoxylin and eosin staining, Verhoeff's van Gieson staining, ELISA, and immunohistochemical analysis were performed to detect the effect of melatonin in vivo.

**Results:**

Melatonin suppressed cell apoptosis directly reduced the expression of endothelial cell damage markers in HCAECs, and alleviated vasculitis in the CAWS‐induced KD mouse model. Mechanistically, melatonin promoted autophagy by activating the melatonin/ melatonin receptor (MT)/cAMP‐response element binding protein (CREB) pathway and upregulating the expression of autophagy‐related gene‐3, thereby suppressing cell apoptosis in an autophagy‐dependent manner. Additionally, melatonin decreased the production of pro‐inflammatory cytokines in macrophages and indirectly reduced the immunopathological damage of HCAECs.

**Conclusions:**

This study revealed that melatonin protects vascular endothelial cells in KD, by suppressing cell apoptosis in an autophagy‐dependent manner and reducing the immunopathological damage mediated by macrophages.

## Graphical abstract

KD‐related vasculitis is a crosstalk between vascular endothelial cells and immune cells, especially macrophages. Macrophages release pro‐inflammatory cytokines to mediate the damage of vascular endothelial cells, when the latter is injured, it can promote the former to secrete more pro‐inflammatory cytokines by releasing damage‐associated molecular pattern. Melatonin alleviates vascular endothelial cells injury directly by suppressing apoptosis in an autophagy‐dependent manner, it also decreases the production of pro‐inflammatory cytokines released by macrophages and reduces the immunopathological damage of vascular endothelial cells in KD‐related vasculitis.

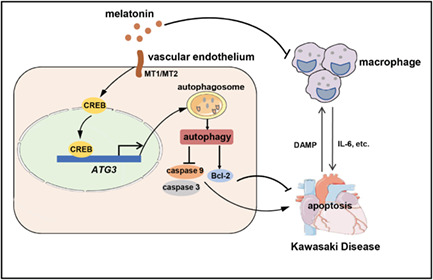

## 1 INTRODUCTION

Kawasaki disease (KD) is one of the most common childhood vasculitis that predominantly affects children younger than 5 years of age, and is the leading cause of childhood‐acquired heart disease in developed countries.^1^, ^2^, ^3^, ^4^ Coronary artery aneurysms (CAAs) are one of the most lethal complications of KD. Despite four decades of investigation, the cause and pathogenesis of KD remain unclear, and current evidence suggests that genetics, inflammation, and autoimmunity may be responsible for the occurrence of the disease.^5^ Although timely treatment with intravenous immunoglobulin (IVIG), which is regarded as the mainstay therapy, has been shown to reduce the incidence of CAA,^6^ a considerable number of patients do not respond to IVIG therapy, which places them at an increased risk of coronary complications.^7^ In addition, other approaches, including a second round of IVIG, glucocorticoid steroids,^8^ infliximab, etc., have also not been found to substantially reduce the residual risk of CAA.^6^ Therefore, there is an urgent need to explore additional therapies to reduce the incidence of CAA in KD, and to alleviate the burden of the disease.

Melatonin (N‐acetyl‐5‐methoxytryptamine) is an endogenous chemical hormone that is mainly synthesized in the pineal gland and released into the circulation following a circadian rhythm. Melatonin functions in diverse ways and presents a wide spectrum of physiological and pathophysiological effects, such as regulation of circadian rhythms,^9^ immune regulation,^10^ antioxidant,^11^ and prevention of cancer metastasis.^12^ Generally, melatonin functions by binding to the G protein‐coupled receptors (GPCRs) MT1 and MT2 (melatonin receptor 1 and 2), both of which are expressed in almost all nucleate cells.^13^ GPCRs play an important pathophysiological role in the occurrence and development of diseases through their interaction with downstream signal transduction molecules. Melatonin has been reported to protect against cardiovascular disease‐mediated myocardial damage, including ischemia/reperfusion,^14^ atherosclerosis,^15^ hypertension,^16^, ^17^ and thoracic aortic aneurysm and dissection.^11^ However, the role of melatonin in KD remains unclear.

Autophagy is an essential process for degrading misfolded proteins or damaged organelles, to maintain cellular homeostasis.^18^ With the help of a series of dynamic membrane‐rearrangement reactions mediated by autophagy‐related gene (ATG) products,^19^ unnecessary cellular components, including misfolded proteins, damaged organelles, and invading pathogens, are delivered to the lysosome by double‐membraned vesicles called autophagosomes, for degradation.^19^ Accumulating evidence suggests that increased autophagy attenuates vasculitis in various cardiovascular diseases.^20^, ^21^ Intriguingly, studies have shown that some existing modality therapies may attenuate KD via the induction of autophagy. Huang et al. demonstrated that the mRNA levels of autophagy markers including microtubule‐associated protein 1 light chain (*LC)3B*, *BECLIN1*, and *ATG16L1* were downregulated in the white blood cells of children with KD, but these increased significantly after IVIG therapy.^22^ Another study showed that resveratrol may improve the inflammatory response of coronary arteries in KD, by exerting anti‐inflammatory effects on human coronary artery endothelial cells (HCAECs), via induction of autophagy.^23^

Apoptosis is the predominant form of programmed cell death. Physiological apoptosis aids in maintaining cellular homeostasis, whereas pathological apoptosis leads to diseases. Autophagy and apoptosis control the turnover of cellular proteins and organelles, and the two are generally negatively correlated. Autophagy promotes cell survival and blocks apoptosis. However, autophagy has also been reported to promote apoptosis in some specific conditions, such as tumours.^24^, ^25^ Autophagy and apoptosis interact with each other by sharing some proteins, such as the Beclin1/B‐cell lymphoma‐2 (Bcl‐2) complex. Beclin 1 is an essential protein that regulates autophagy by interacting with the anti‐apoptotic protein Bcl‐2.^25^, ^26^ Apoptosis caused by excessive immune activation and oxidative stress leads to vascular injury in KD patiens,^27^, ^28^ while autophagy has been shown to protect endothelial cells by attenuating inflammation in a KD mouse model.^21^ Nevertheless, the role of the interaction between endothelial cell autophagy and apoptosis in the pathogenesis of KD remains uncertain.

Considering the protective roles of melatonin and autophagy in a series of cardiovascular and autoimmunity diseases,^29^ in the present study, we aimed to investigate the function of melatonin in the vasculitis of KD and determine whether the underlying mechanism is autophagy‐dependent.

## 2 MATERIAL AND METHODS

### 2.1 Human subjects

A total of 16 patients diagnosed with KD (10 boys and 6 girls; mean age = 2.9 years) were recruited between July 2020 and October 2020, from the Cardiovascular Center of Children's Hospital of Fudan University, Shanghai, China. KD diagnosis was based on the guidelines released by the American Heart Association^6^ and the Japanese Circulation Society.^30^, ^31^ The flow chart of KD patient selection process is shown in Figure S1. Peripheral blood mononuclear cells (PBMCs) from these patients were isolated using Ficoll‐Paque density gradient solution (GE Healthcare, USA). Briefly, peripheral blood was centrifuged at 400 *g* for 10 min, to separate the supernatant serum, while the sedimented blood cells were mixed with phosphate‐buffered saline (PBS) and overlaid on top of Ficoll. Post centrifugation at 400 *g* for 30 min, the PBMCs were aspirated from the intermediate interface. The cell pellet was washed twice with PBS, and the CD14^+^ cells obtained from the PBMCs were sorted using a magnetic cell sorting system (Miltenyi Biotec, Germany) and then cultured in Roswell Park Memorial Institute (RPMI)‐1640 medium (Gibco, USA) containing 10% fetal bovine serum (FBS; Gibco), 1% penicillin/streptomycin (Gibco), and 10 ng/ml recombinant human macrophage colony‐stimulating factor‐1 (rhM‐CSF, R&D Systems, USA) for 7 days, to generate the macrophages. The macrophages were then stimulated with 100 ng/ml lipopolysaccharide (LPS, Sigma‐Aldrich, USA) or 20 ng/ml recombinant human tumour necrosis factor‐α (TNF‐α, R&D Systems), with or without 0.5 mM melatonin (Sigma‐Aldrich) pre‐treatment.

Written informed consent was obtained from all the participants. The study was approved by the Research Ethics Board of the Children's Hospital of Fudan University (IRB protocol number: 2019‐328). The inclusion criteria for KD patients in this study were as follows: (i) fulfilled the diagnostic criteria of the guidelines released by the Japanese Circulation Society^30^, ^31^ and American Heart Association^6^; (ii) were in the timeframe of within 5–10 days of illness and had not yet been treated with IVIG, aspirin, steroids, or immunosuppressive drugs; and (iii) the patients and their guardians voluntarily participated in this study and signed the informed consent form. The exclusion criteria for KD patients in this study were as follows: (i) did not fulfil the inclusion criteria; (ii) had a previous history of KD; (iii) had severe concomitant medical disorders; and (iv) did not have samples available for collection. Blood samples were collected on the day of admission prior to the administration of IVIG, aspirin, steroids, or immunosuppressive drugs. The general patient information is listed in Table S1. The time of sample collection, treatment, and representative laboratory data before IVIG treatment are shown in Table S2. The urinary levels of 6‐sulfatoxymelatonin in the patients are shown in Table S3.

### 2.2 Cell culture, stimulation, and transfection

HCAECs were purchased from ScienCell Research Laboratories (USA) and cultured in flasks coated with fibronectin (2 μg/cm^2^), in a humidified atmosphere containing 5% CO_2_ at 37°C. The medium was an endothelial cell medium (ECM, ScienCell) containing 1% endothelial cell growth supplement, 5% FBS, and 1% penicillin/streptomycin. To investigate the effect of melatonin, the cells were incubated in ECM containing 0.5 mM melatonin for 6 h, and then stimulated with 20 ng/ml TNF‐α for different time points. Transient transfections of small interfering RNAs (siRNAs; GenePharma, China) were performed using Lipofectamine® RNAiMAX reagent (Invitrogen, USA), while those of plasmids were performed using Lipofectamine® 2000 (Invitrogen), according to standard procedures. The sequences of the siRNAs are listed in Table S4.

The human THP1 cell line was obtained from American Type Culture Collection (USA) and maintained in RPMI‐1640 medium with inactivated 10% FBS and 1% penicillin–streptomycin. THP1 cells were induced into macrophages with 200 ng/ml phorbol 12‐myristate 13‐acetate (Sigma‐Aldrich) for 48 h. LPS (100 ng/ml) or TNF‐α (20 ng/ml) was then used to induce macrophage activation, with or without 0.5 mM melatonin (Sigma‐Aldrich) pre‐treatment.

### 2.3 Co‐culture of HCAECs and THP1 cells

After stimulation with LPS (100 ng/ml), the culture supernatant of the THP1‐derived macrophages, with or without melatonin pre‐treatment, was collected and centrifuged for 30 min at 3000 rpm, 4°C, to remove the cell debris. After HCAECs were seeded into 6‐well or 96‐well cell culture plates for 24 h, the ECM medium was replaced with a mixed conditional medium containing a 3:1 volume ratio of ECM to THP1 culture supernatant. Cell proliferation and apoptosis were detected at the designated time‐points.

### 2.4 Cell proliferation assay

Cell proliferation was detected using the Cell Counting Kit‐8 (CCK8) Assay Kit (Dojindo, Japan), according to the manufacturer's instructions. Briefly, HCAECs were seeded into 96‐well plates at a density of 2000 cells/well. After the corresponding treatment, cell viability was detected at the designated time‐points (0, 24, 48, and 72 h), by adding 10 μl CCK8 solution to the cells and incubating for 2 h at 37°C. The absorbance at 450 nm was measured using a microplate reader (BioTek, USA).

### 2.5 Cell apoptosis assay

Apoptosis was examined using an Annexin V‐ fluorescein isothiocyanate (FITC)/propidium iodide (PI) double‐staining kit (BD Biosciences, USA), following the manufacturer's protocol. HCAECs were cultured into 6‐well plates (2 × 10^5^ cells/well) for 48 h. After melatonin and TNF‐α treatment, the cells were digested with ethylenediaminetetraacetic acid (EDTA)‐free trypsin. Next, the cells were stained with FITC and PI, and subjected to flow cytometry using FACSCanto™ II (BD Biosciences). The data were analysed using FlowJo V10 software (Tree Star, USA).

### 2.6 RNA extraction and reverse transcription quantitative PCR (RT‐qPCR)

Total RNA was extracted from the cells using TRIzol® reagent (Invitrogen) and reverse transcribed into complementary DNA (cDNA) using the PrimeScript RT Reagent Kit (Takara Bio, Japan), according to the manufacturer's instructions. To quantify mRNA expression, cDNA was amplified using qPCR with the SYBR® Premix Ex Taq™ RT‐PCR Kit (Takara Bio) on a Roche 480 Real Time PCR System (Roche, Switzerland). β‐actin was used as an internal reference, and the relative expression was calculated using the 2^−ΔΔCT^ method. The primer sequences used in this study are provided in Table S5.

### 2.7 Transwell assay

THP1‐derived macrophages were added to the top chamber of polycarbonate Transwells (Corning, USA), while HCAECs (pre‐treated with or without 0.5 mM melatonin) were seeded in the bottom chamber and stimulated with 20 ng/ml TNF‐α. After co‐culturing for 24 h in a 5% CO_2_‐containing atmosphere at 37°C, the macrophages on the upper membrane surface were wiped gently with cotton swabs and washed with cold PBS. Cells on the underside of the membrane were fixed with 4% paraformaldehyde and stained with Crystal Violet. Next, the number of migrated macrophages on the membrane underside was counted using a microscope (Leica, Germany), in five random fields.

### 2.8 Western blot

Proteins from cells lysed in radioimmunoprecipitation assay buffer containing protease inhibitors were quantified using a bicinchoninic acid protein assay kit (Beyotime, China) and then denatured at 100°C for 10 min. Proteins (15 μg/lane) were separated using sodium dodecyl sulfate‐polyacrylamide gel electrophoresis (SDS‐PAGE). Cleaved caspase‐9, Bcl‐2, β‐actin, protein kinase B (AKT), phosphorylated AKT (p‐AKT), and p62 were separated using 10% SDS‐PAGE; cleaved caspase‐3, LC3‐I, and LC3‐II were separated using 12% SDS‐PAGE; mammalian target of rapamycin (mTOR) and phosphorylated mTOR (p‐mTOR) proteins were separated using 8% SDS‐PAGE. The proteins were then transferred onto polyvinylidene difluoride membranes (Bio‐Rad, USA), and blocked with 5% bovine serum albumin (BSA, Bio‐Rad) in Tris‐buffered saline (TBS, Bio‐Rad) with 0.1% Tween‐20 (Sigma‐Aldrich). The membranes were incubated with primary antibodies against LC3‐II/I, p62, AKT, p‐AKT, mTOR, p‐mTOR, cleaved caspase‐9, cleaved caspase‐3, Bcl‐2 (1:1000, Cell Signalling Technology, USA), and β‐actin (1:1000, Cell Signalling Technology) diluted in blocking buffer at 4°C overnight. After three washes with TBS containing 0.1% Tween‐20, the membranes were incubated with horseradish peroxidase‐conjugated secondary antibodies (1:2000, ProteinTech, USA) for 1 h at room temperature. An enhanced chemiluminescent western blotting substrate kit (Thermo Fisher Scientific, USA) was used for detection. The signals were imaged with a ChemiDoc™ XRS+ Imager (Bio‐Rad). The expression level of β‐actin served as an internal control for protein loading.

### 2.9 Enzyme‐linked immunosorbent assay (ELISA)

TNF‐α and interleukin (IL)‐6 levels in mouse serum were measured using mouse TNF‐α and IL‐6 ELISA kits (MultiSciences, China). IL‐6 levels in the cultured supernatants of HCAECs and THP1‐derived macrophages were detected using a Human IL‐6 ELISA Kit (R&D Systems). The melatonin metabolite 6‐sulfatoxymelatonin in the urine of mice was measured using a Melatonin‐Sulfate Urine ELISA kit (IBL, Germany). Melatonin metabolite 6‐sulfatoxymelatonin in the urine of KD patients was also measured using a Melatonin‐Sulfate Urine ELISA kit (IBL). All assays were performed according to the manufacturer's instructions.

### 2.10 Autophagic flux assay

For the kinetics of autophagic flux, HCAECs were infected with an adenoviral vector expressing monomeric red fluorescent protein (mRFP)‐green fluorescent protein (GFP)‐LC3 (HanBio Technology, China), according to the manufacturer's instructions. After the corresponding treatment, the cells were washed with PBS, fixed with 4% paraformaldehyde, sealed with 4′,6‐Diamidine‐2′‐phenylindole dihydrochloride (DAPI) (Biotime, China), and viewed under a fluorescence microscope (Eclipse E800, Nikon, Japan). The number of GFP and mRFP dots was determined by manually counting the fluorescent puncta in five fields from three different cell preparations, in a 40× visual field. The nuclear number was evaluated by counting the number of DAPI‐stained nuclei in the same field. The average number of dots per cell was obtained by dividing the total number of dots by the number of nuclei in each microscopic field.

### 2.11 RNA sequencing (RNA‐seq)

HCAECs stimulated with TNF‐α (20 ng/ml), with or without melatonin (0.5 mM) pre‐treatment, were prepared for RNA‐seq (Sinotech Genomics, China). RNA extraction was performed, followed by the assessment of integrity using an Agilent Bioanalyzer 2100 (Agilent Technologies, USA), and measurement of the concentration and purity using a Qubit® 3.0 Fluorometer (Life Technologies, USA) and NanoDrop™ One spectrophotometer (Thermo Fisher Scientific). Next, the samples were sequenced using next‐generation sequencing on an Illumina sequencing platform (Novaseq 6000, Illumina, USA). After sequencing, the filtered high‐quality sequences (clean data) filtered from the original raw data were mapped to the *Homo sapiens* GRCh38 genome. Based on the mapped results, the expression level of each gene was calculated, and the samples of the control and melatonin groups were subjected to further analysis such as expression difference, enrichment, and cluster analysis. Genes with *p* ≤ 0.05 and |fold change| ≥ 2.0 were considered significantly differentially expressed. The data referred to in this study have been deposited in Gene Expression Omnibus database, with the accession number GSE183359.

### 2.12 Plasmid construction

The full‐length cAMP‐response element binding protein (CREB) coding DNA sequence (CDS) was inserted into the blank pCMV‐C‐Flag plasmid at HindIII and XbaI restriction sites, to construct the CREB‐CDS vector. The *ATG3* promoter sequence containing CERB binding sites was inserted into the pGL3‐basic plasmid at NheI and XhoI restriction sites, to generate the *ATG3*‐promoter plasmid. The pRL‐Tk Renilla plasmid was purchased from Tsingke Biological (China). The primer pairs for the amplification of full‐length *CREB* and *ATG3* promoters are provided in Table S6.

### 2.13 Dual‐luciferase reporter assay

293 T cells were seeded into 96‐well plates, followed by co‐transfection of *CREB*‐CDS, *ATG3*‐promoter, and Renilla plasmids using Lipofectamine™ 2000 transfection reagent, according to the manufacturer's protocol. After 48 h, the luciferase activity in the cells was analysed using the Dual‐Luciferase® Reporter Assay System (Promega, USA).

### 2.14 Preparation of *Candida albicans* water‐soluble fraction (CAWS)

CAWS was prepared from *C. albicans* strain NBRC1358 (NITE Biological Resource Center, Japan), as previously described.^5^, ^27^ Briefly, *C. albicans* was cultured in C‐limiting medium at 400 rpm, 27°C, for 48 h. Following culture, an equal volume of ethanol was added, and the mixture was incubated overnight. The precipitate was then collected, dissolved in 250 ml of distilled water, and an equal volume of ethanol was added to it. This mixture was left to stand overnight, following which the precipitate was collected and dried with acetone to obtain CAWS.

### 2.15 CAWS‐induced KD mouse model and animal grouping

All experimental procedures were approved by the Local Ethics Committee of the Children's Hospital of Fudan University, and all animals received humane care, according to the Guidelines for Ethical Care of Experimental Animals of Children's Hospital of Fudan University. C57BL/6 wild‐type mice (4 weeks of age, male) were purchased from SLAC Laboratory Animal Co. Ltd. (China) and maintained in a specific pathogen‐free room at 22 ± 2°C, with automatic light cycles (12/12 h light/darkness) and 50% humidity.

The mice were divided into three equal experimental groups (16 mice/group). Mice in the PBS group were intraperitoneally (i.p.) injected with 0.1 ml PBS for 30 days, from day −2; mice in the CAWS group received an i.p. dose of 8 mg CAWS daily, for 5 consecutive days, from day 0; and mice in the CAWS+melatonin group received 8 mg i.p. CAWS, daily for 5 consecutive days, from day 0, as well as, i.p melatonin for 30 days, from day −2. Melatonin (dissolved in absolute ethanol and diluted with PBS before use) was administered at a dose of 40 mg/kg/day. Mice (*n* = 8) in each group were anaesthetized and sacrificed on days 14 and 28, following which their blood samples were collected. The serum samples were separated by means of centrifugation at 2000 rpm for 10 min, and then stored at −80°C until analysis. Part of the heart and abdominal aorta tissues were isolated and immediately frozen in liquid nitrogen until required for molecular biology experiments, while the rest were fixed with 4% paraformaldehyde for pathological examination.

For the construction of the *Atg*3‐knockdown mouse model, C57BL/6 wild‐type mice (4 weeks of age, male) were infected with a lentivirus vector carrying short hairpin RNA (shRNA; GenePharma), according to the manufacturer's instructions. Mice in the si‐*Atg3+*CAWS+melatonin group received 5 × 10^7^ transducing units (TU) of si‐*Atg3* lentivirus by means tail intravenous injection on day −3, followed by 8 mg i.p. CAWS, for 5 consecutive days, from day 0, as well as, i.p. melatonin, for 30 days, from day −2. Meanwhile, mice in the negative control group (si‐NC+CAWS+melatonin) received 5 × 10^7^ TU of negative control lentivirus, with the administration of CAWS and melatonin being the same as that in the si‐*Atg3*+CAWS+melatonin group. The sequences of shRNAs carried by the lentivirus are listed in Table S7.

### 2.16 Histological analysis

The fixed hearts and abdominal aortas were embedded in paraffin and sectioned. Step sections in the horizontal direction were made every 10 μm, to observe the histological changes in the coronary artery, aortic root, and abdominal aorta in detail. Haematoxylin and eosin (H&E) staining was performed to observe the inflammatory changes, by means of light microscopy. Verhoeff's van Gieson (EVG) staining was used to investigate the vascular architecture. Immunohistochemical analysis was performed to detect the expression level of Bcl‐2 using an anti‐mouse Bcl‐2 antibody (1:200, Cell Signalling Technology). The stained sections were photographed under a light microscope (Leica).

### 2.17 Statistical analysis

All experiments were repeated at least three times to guarantee robust and unbiased results. Continuous variables are presented as mean ± standard deviation. Comparisons between two groups were performed using the two‐tailed Student's *t*‐test, while multi‐group comparisons were carried out using one‐way analysis of variance. Statistical significance was set at *p* ≤ 0.05; *p < 0.05, **p < 0.01, and ***p < 0.001. Statistical analyses were performed using SPSS version 19.0 (International Business Machines Corporation, USA), and data were visualized using Prism software version 9.0 (GraphPad Software, USA).

## 3 RESULTS

### 3.1 Melatonin alleviates CAWS‐induced KD‐like vasculitis in mice

**FIGURE 1:**
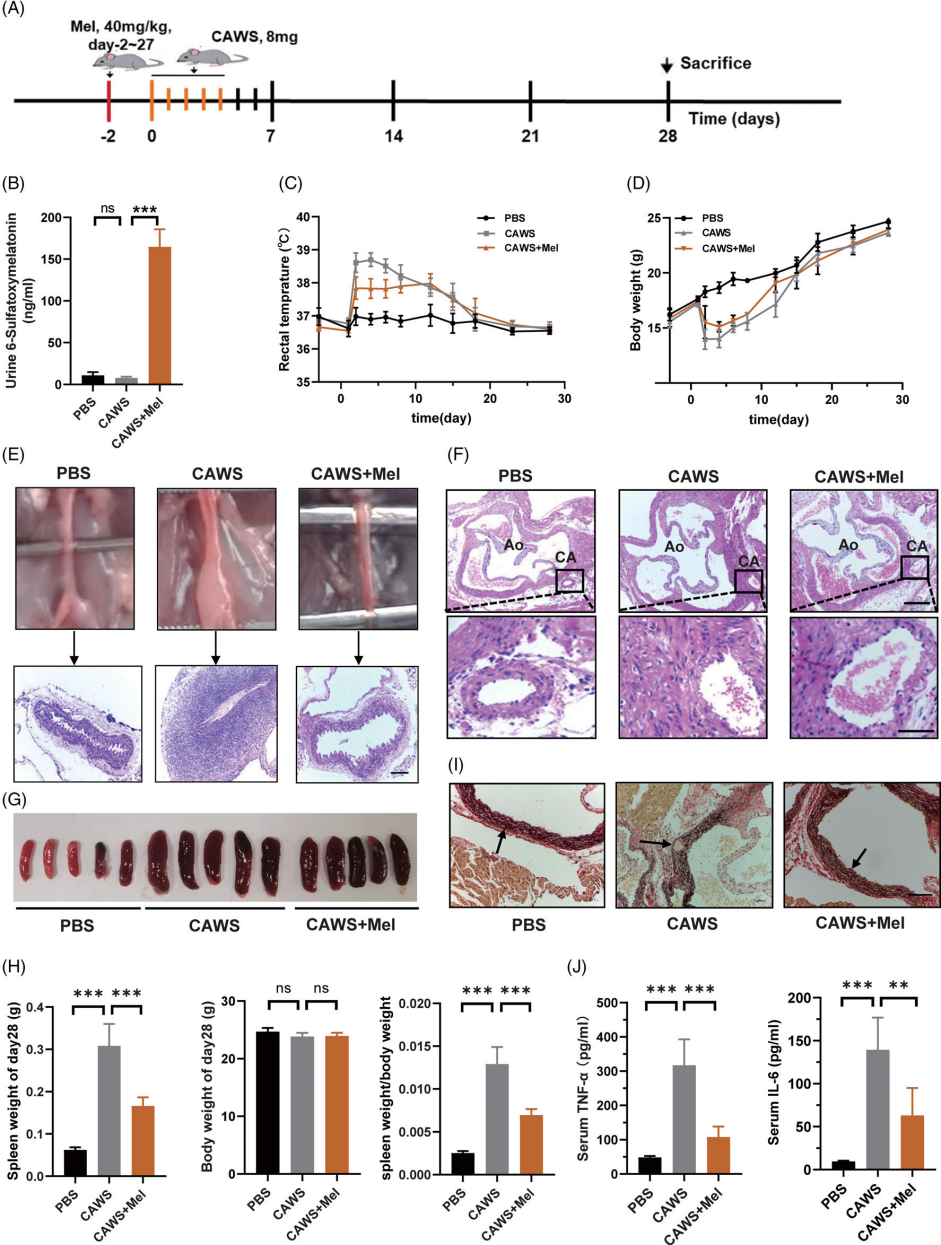
Melatonin alleviates CAWS‐induced KD‐like vasculitis in mice. (A) Modelling timeline of the mouse model. C57BL/6 mice in the CAWS group received i.p. injection of 8 mg CAWS for five consecutive days, while mice in the CAWS+Mel group received i.p. injection of 8 mg CAWS for five consecutive days and that of melatonin (40 mg/kg) for 30 days, and mice in the PBS group received were i.p. injection of 0.1 ml PBS at the same time. (B) Concentration of melatonin metabolite 6‐sulfatoxymelatonin in mice urine, as analysed using ELISA. (C) Mean daily rectal temperature change and (D) mean daily body weight change of mice in the three groups. (E) Histopathological changes in the abdominal aorta were examined using H&E staining, and representative images of the abdominal aorta cut on a transverse plane are shown. Scale bar: 200 μm. (F) Representative H&E images of the aortic root and coronary arteries cut on a transverse plane. Scale bar of the upper pictures is 200 μm and that of the lower pictures is 50 μm. (G) Representative images of the mice spleens on day 28. (H) Spleen weights, body weights, and the ratio of the two on day 28. (I) Representative images of Verhoeff's van Gieson staining of the aortic root on day 28, where arrows indicate the vascular elastic fibre. Scale bar: 100 μm. (J) Serum TNF‐α and IL‐6 concentrations of the mice on day 14, as analysed using ELISA. Data are presented as mean ± SEM of three independent experiments. **p < 0.01, ***p < 0.001; ns, not significant. CA, coronary artery; Ao, aorta; CAWS, *Candida albicans* water‐soluble fraction; Mel, melatonin; PBS, phosphate‐buffered saline

Melatonin has been reported to have a protective effect on the vascular endothelium, and we hypothesized that it can also alleviate vasculitis in KD. To investigate the effect of melatonin on KD‐related vasculitis, we first constructed a CAWS‐induced vasculitis mouse model that resembles the typical vascular pathology of KD, as manifested by fever, weight loss, vascular inflammation, and splenomegaly,^5^, ^32^, ^33^ and then treated the mice with melatonin or PBS, according to the modelling timeline (Figure 1A). The results showed that the melatonin metabolite 6‐sulfatoxymelatonin in the urine of mice in the melatonin‐treated group was significantly higher than that in the other groups (Figure 1B). Melatonin treatment significantly relieved CAWS‐induced fever and weight loss, as compared to PBS treatment (Figure 1C,D). Abdominal aortitis was alleviated after melatonin treatment (Figure 1E). H&E staining showed that severe inflammatory cell infiltration was induced by CAWS around the aortic root and coronary artery, which was significantly improved after melatonin treatment (Figure 1F). An improvement in splenomegaly was also observed after melatonin treatment (Figure 1G,H). EVG staining showed that the vascular elastic fibre injury of the aortic root was improved in the melatonin group (Figure 1I). In addition, ELISA assay showed remarkably decreased serum levels of TNF‐α and IL‐6 in the melatonin group, as compared to those in the CAWS group (Figure 1J).

### 3.2 Melatonin inhibits vascular endothelial cell apoptosis

**FIGURE 2:**
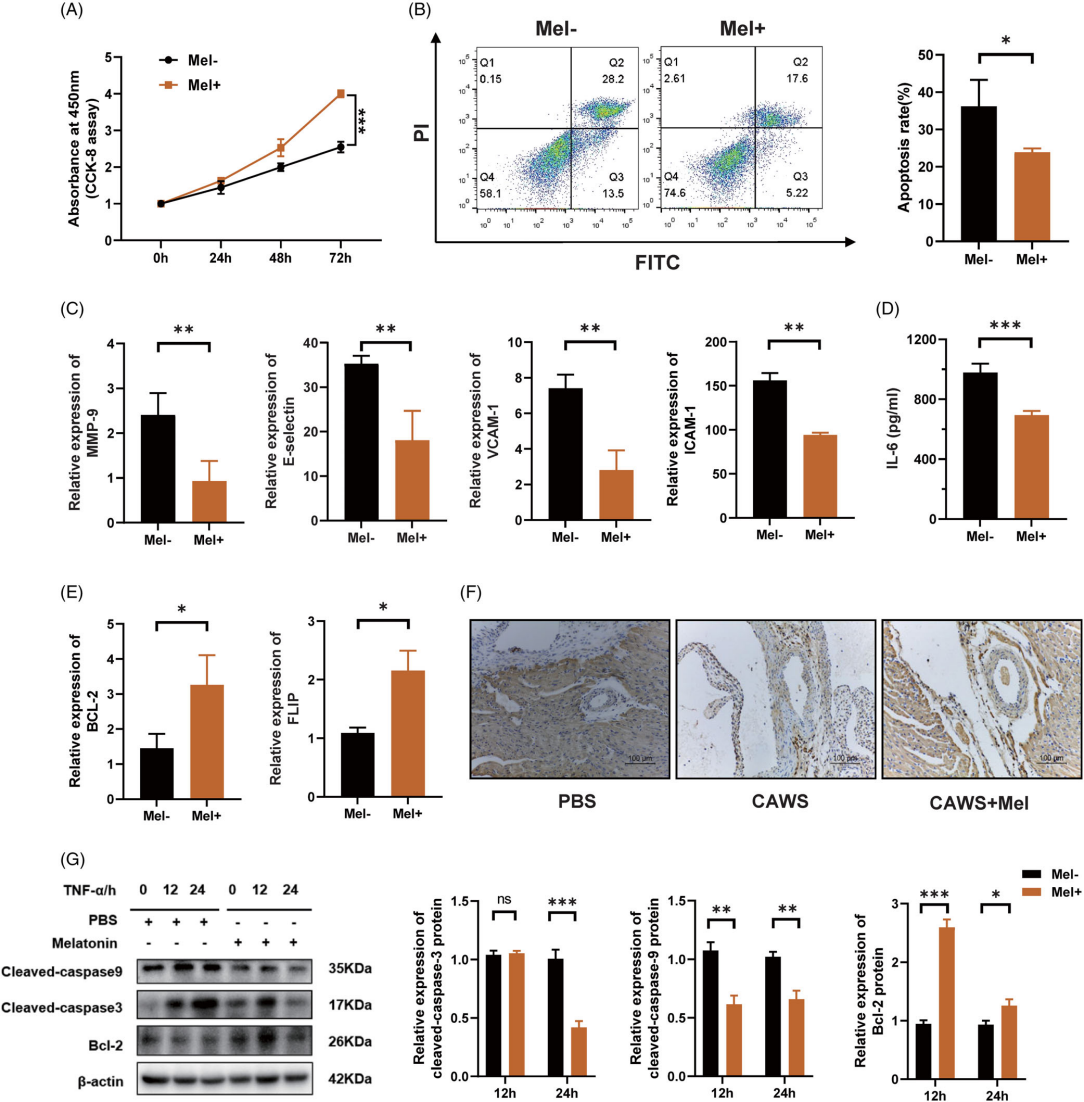
Melatonin inhibits vascular endothelial cell apoptosis. (A) Cell proliferation of HCAECs, as assessed using CCK‐8 assay; the Mel+ group received 0.5 mM melatonin pre‐treatment for 6 h, while the Mel‐ group did not. (B) After pre‐treatment with or without 0.5 mM melatonin for 6 h, the HCAECs were cultured in ECM with H_2_O_2_, to induce cell apoptosis, following which apoptosis was analysed by means of Annexin V‐FITC/PI double‐staining and flow cytometry. (C) Mel+ group was pre‐treated with 0.5 mM melatonin for 6 h, following which both the groups were stimulated with 20 ng/ml TNF‐α for 12 h and relative mRNA expression levels of endothelial cell damage markers, including *MMP‐9*, *E‐selectin*, *ICAM‐1*, and *VCAM‐1* were detected using RT‐qPCR. (D) ELISA assay of the protein level of IL‐6 in cell culture supernatants of HCAECs. (E) The Mel+ group was pre‐treated with 0.5 mM melatonin for 6 h, following which both the groups were stimulated with 20 ng/ml TNF‐α for 12 h and relative mRNA expression levels of anti‐apoptotic genes *Bcl2* and *FLIP* were detected using RT‐qPCR. (F) Immunohistochemical staining of anti‐apoptotic protein Bcl‐2 in mice coronary arteries. Scale bar: 100 μm. (G) The levels of Bcl‐2, cleaved‐caspase‐3, and cleaved‐caspase‐9 in HCAECs stimulated with TNF‐α, with or without melatonin pre‐treatment, were quantified using western blot. Data are presented as mean ± SEM. *p < 0.05, **p < 0.01, and ***p < 0.001; ns, not significant. Bcl‐2, B‐cell lymphoma‐2; CCK, cell counting kit; ECM, endothelial cell medium; FITC, fluorescein isothiocyanate; HCAEC, human coronary artery endothelial cell; ICAM‐1, intercellular cell adhesion molecule‐1; Mel, melatonin; MMP‐9, matrix metalloproteinase 9; PI, propidium iodide; PLIP, FLICE inhibitory protein; TNF‐α, tumour necrosis alpha; VCAM‐1, vascular cell adhesion molecule‐1

Next, we investigated whether melatonin plays a direct role in protecting vascular endothelial cells. To answer this question, we performed proliferation and apoptosis assays on HCAECs, with or without melatonin treatment. Subsequent results showed that cell proliferation was robustly promoted (Figure 2A), while apoptosis was significantly inhibited (Figure 2B) upon melatonin treatment. Vascular endothelial cell damage was also inhibited by melatonin, manifested as significantly decreased mRNA levels of endothelial cell damage markers, including matrix metalloproteinase 9 (*MMP‐9*), *E‐selectin*, intercellular cell adhesion molecule‐1 (*ICAM‐1*), and vascular cell adhesion molecule‐1 (*VCAM‐1*) (Figure 2C). ELISA revealed a significant reduction in the protein level of IL‐6 in the cell culture supernatant of HCAECs (Figure 2D). In addition, the mRNA levels of anti‐apoptotic genes, including *BCL2* and FLICE inhibitory protein (*FLIP*), were significantly increased after melatonin treatment (Figure 2E). Immunohistochemical staining showed that the anti‐apoptotic protein Bcl2 was significantly reduced in the vascular walls of CAWS‐treated mice, but was remarkably elevated after the administration of melatonin (Figure 2F). Consistently, western blot showed that the expression of Bcl‐2 increased, while those of the pro‐apoptotic proteins, including cleaved‐caspase‐3 and cleaved‐caspase‐9, decreased after melatonin treatment (Figure 2G). Collectively, these results suggested that melatonin can inhibit vascular endothelial cell apoptosis.

### 3.3 Melatonin promotes autophagy via the phosphatidylinositol‐3‐kinase (PI3K)/AKT/mTOR signalling pathway in HCAECs

**FIGURE 3:**
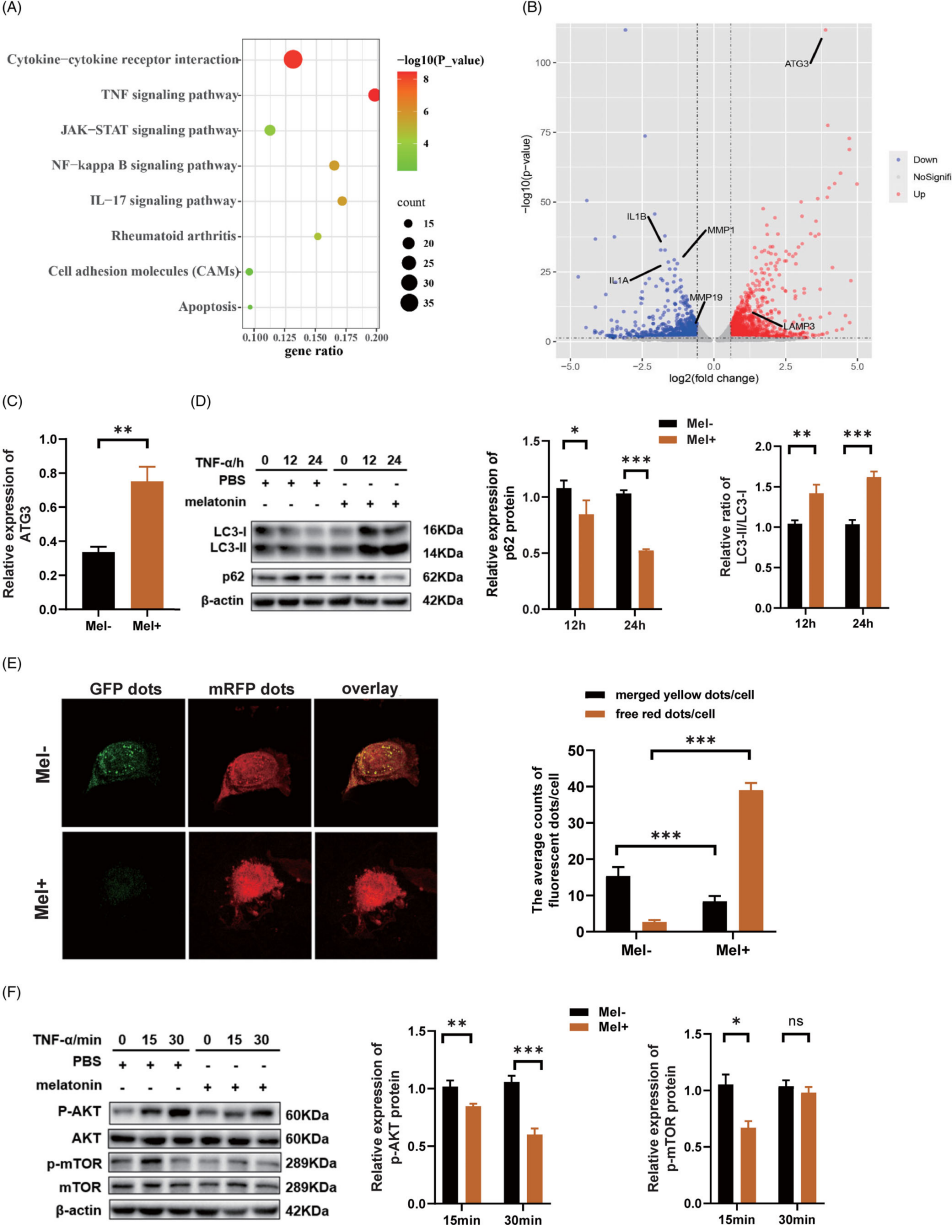
Melatonin promotes autophagy via the PI3K/ATK/mTOR signalling pathway in HCAECs. (A) KEGG analysis showing the enriched pathways of the differentially expressed genes in HCAECs stimulated with TNF‐α (20 ng/ml, 12 h), with or without melatonin pre‐treatment (0.5 mM, 6 h). (B) Genes related to autophagy were upregulated, while those related to apoptosis and vascular endothelial cell damage were downregulated, after melatonin treatment. (C) Relative expression of *ATG3* after melatonin (0.5 mM, 6 h) pre‐treatment and TNF‐α (20 ng/ml, 12 h) stimulation, as assessed using RT‐qPCR. (D) The protein levels of LC3‐II, LC3‐I, and p62 were quantified using western blot. (E) HCAECs were infected with an adenoviral vector expressing mRFP‐GFP‐LC3, and the kinetics of autophagic flux was detected using a fluorescence microscope. (F) Western blot showing the protein levels of AKT, p‐AKT, mTOR and p‐mTOR in HCAECs stimulated with TNF‐α (20 ng/ml), with or without melatonin pre‐treatment (0.5 mM, 6 h). Data are presented as mean ± SEM. *p < 0.05, **p < 0.01, and ***p < 0.001; ns, not significant. AKT, protein kinase B; *ATG3*, autophagy‐related gene‐3; GFP, green fluorescent protein; KEGG, Kyoto Encyclopedia of Genes and Genomes; LC3, microtubule‐associated protein 1 light chain 3; mRFP, monomeric red fluorescent protein; mTOR, mammalian target of rapamycin

To explore the mechanism and target of melatonin's protective effect on HCAECs, we performed RNA‐seq on TNF‐α‐stimulated HCAECs, with or without melatonin pre‐treatment. Kyoto Encyclopedia of Genes and Genomes (KEGG) pathway analysis revealed that differentially expressed genes were mainly enriched in signalling pathways related to inflammation, cytokine interaction, autophagy, and apoptosis (Figure 3A). Genes related to autophagy were remarkably upregulated while those related to apoptosis and vascular endothelial cell damage were downregulated after melatonin treatment (Figure 3B). Among these differentially expressed genes, *ATG3*, an autophagy‐related gene, attracted our attention. We further verified the sequencing data in HCAECs using RT‐qPCR, and the results showed that the expression of *ATG3* was indeed upregulated after melatonin treatment (Figure 3C). Western blot showed that TNF‐α‐stimulated HCAECs with melatonin pre‐treatment exhibited a significant increase in the ratio of LC3‐II/I, which is indicative of autophagic activation, and a significant decrease in p62, whose expression level is negatively correlated with autophagy (Figure 3D), indicating that autolysosome formation was significantly increased in the melatonin group. Moreover, to determine whether melatonin has a robust autophagic flux‐promoting effect on HCAECs under pathological conditions, HCAECs were infected with an adenoviral vector expressing mRFP‐GFP‐LC3, to detect the kinetics of autophagic flux. As expected, a greater number of free red dots were seen in the melatonin group, while a greater number of merged yellow dots were seen in the control group (Figure 3E), suggesting the activation of autophagic flux after melatonin treatment. Furthermore, to understand the signalling pathways involved in the regulation of autophagy by melatonin, we investigated alterations in the PI3K/AKT/mTOR pathway, which is negatively correlated with autophagy activation. Western blot showed that melatonin treatment substantially reduced the levels of p‐AKT and p‐mTOR (Figure 3F). Overall, these results support that melatonin promotes autophagy by inhibiting the PI3K/ATK/mTOR signalling pathway in HCAECs.

### 3.4 MT/CREB/*ATG3* pathway is responsible for melatonin‐repressed apoptosis in HCAECs, in an autophagy‐dependent manner

**FIGURE 4:**
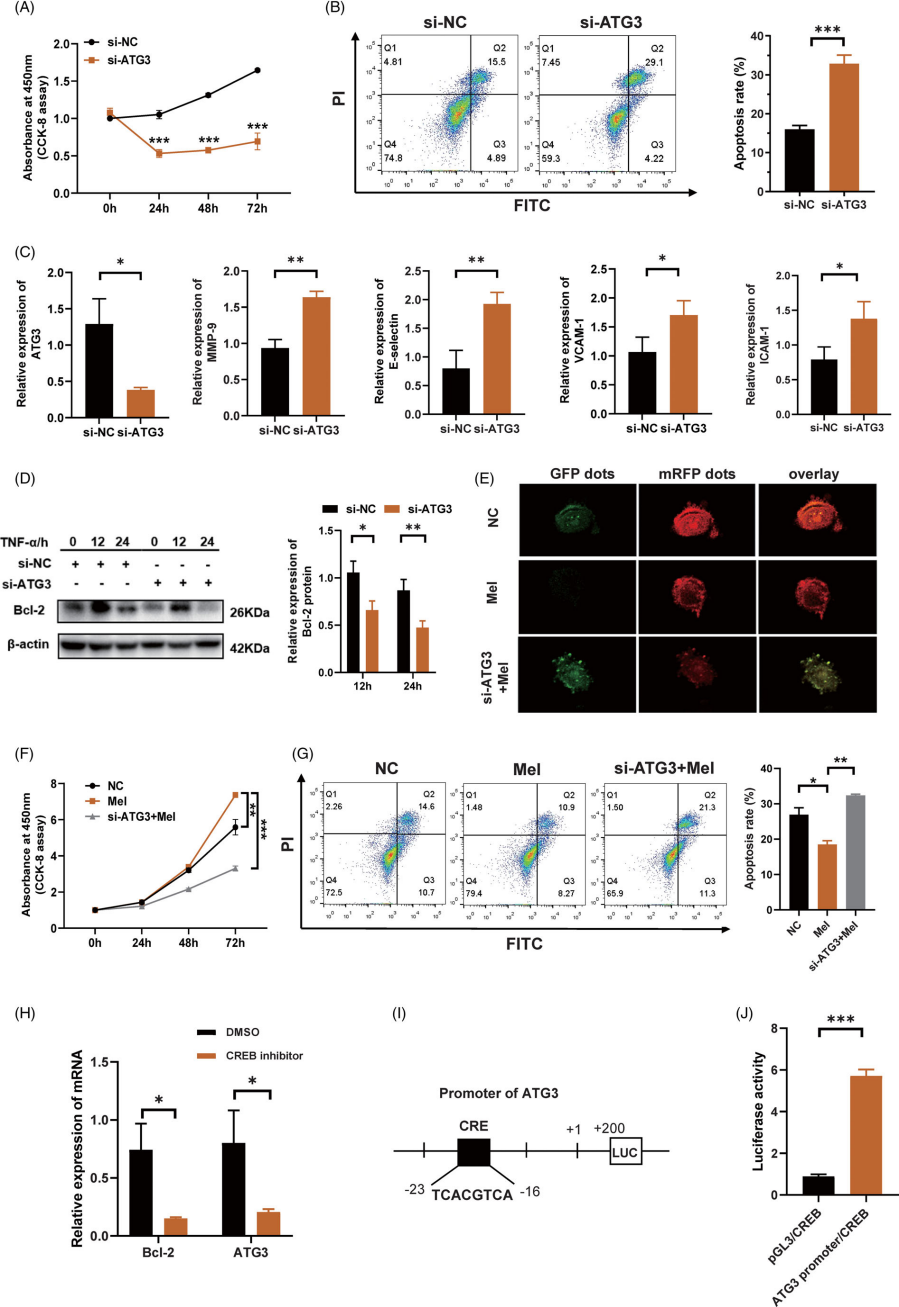
MT/CREB/ATG3 pathway is responsible for melatonin‐repressed apoptosis in HCAECs, in an autophagy‐dependent manner. (A) Cell proliferation of HCAECs, with or without *ATG3* knockdown, as assessed using CCK‐8 assay. (B) Cell apoptosis of HCAECs, with or without *ATG3* knockdown, was examined by means of Annexin V‐FITC/PI double‐staining and flow cytometry. Cell percentages in the Q3 and Q2 quadrants (indicating the fraction of apoptotic cells) were calculated, and are presented as histograms. (C) Relative expression of *ATG3*, *MMP‐9*, *E‐selectin*, *VCAM‐1*, and *ICAM‐1* after *ATG3*‐knockdown in HCAECs, as analysed using RT‐qPCR. (D) Western blot showing the expression of Bcl‐2 in *ATG3‐*deficient HCAECs. (E) Kinetics of autophagic flux was detected using a fluorescence microscope, after the HCAECs were infected with an adenoviral vector expressing mRFP‐GFP‐LC3. (F) Cell proliferation of HCAECs was assessed using CCK‐8 assay. Mel, melatonin group (melatonin, 0.5 mM, 6 h); si‐*ATG3*+Mel, melatonin treatment after silencing of *ATG3*; NC, negative control. (G) Cell apoptosis of HCAECs, as assessed using flow cytometry. (H) Relative expression of *ATG3* in the presence of CREB inhibitor, as analysed using RT‐qPCR. *BCL2* served as a positive reference. (I) *ATG3* promoter sequence with CREB binding site. (J) Luciferase activity of 293 T cells co‐transfected with *CREB*‐CDS vector or vector carrying *ATG3* promoter sequence, or co‐transfected with *CREB*‐CDS vector and blank vector without *ATG3* promoter sequence. Data are presented as mean ± SEM. *p < 0.05, **p < 0.01, and ***p < 0.001; ns, not significant. *ATG3*, autophagy‐related gene‐3; CDS, coding sequence; CREB, cAMP‐response element binding protein; GFP, green fluorescent protein; ICAM‐1, intercellular cell adhesion molecule‐1; MMP‐9, matrix metalloproteinase 9; mRFP, monomeric red fluorescent protein; MT, melatonin receptor; VCAM‐1, vascular cell adhesion molecule‐1

To explore the role of *ATG3* in the protective effect of melatonin in HCAECs, we first transfected cells with an siRNA to silence *ATG3*. The CCK‐8 assay showed a remarkable decrease in cell proliferation (Figure 4A), while flow cytometry showed a significant increase in cell apoptosis after *ATG3* knockdown (Figure 4B). A notable increase in endothelial cell damage markers, including *MMP‐9*, *E‐selectin*, *VCAM‐1*, and *ICAM‐1*, was also observed after *ATG3* knockdown (Figure 4C). As expected, western blot demonstrated that the expression of Bcl‐2 was remarkably reduced in the *ATG3*‐knockdown group, as compared to that in the control group (Figure 4D). To further confirm that melatonin has a protective effect on HCAECs by regulating *ATG3* expression, we performed following experiments. A subsequent autophagic flux kinetics assay showed that the manifestation of more free red dots in the melatonin group was offset in the *ATG3*‐knockdown group, which presented more merged yellow dots (Figures 4E and S2). In addition, *ATG3*‐knockdown counteracted the stimulatory effect of melatonin on cell proliferation (Figure 4F), and the apoptosis‐inhibitory effect of melatonin disappeared after *ATG3* knockdown (Figure 4G). Generally, melatonin binds to its membrane receptors, MT1 and MT2, and initiates downstream signal transduction. To explore the signal transduction molecules that mediate the interaction between MT and *ATG3*, we analysed the transcription factors related to the GPCR pathway and predicted the transcription factors that can bind to the promoter region of *ATG3*. CREB appeared to be a candidate. The upregulation of *ATG3* gene expression by melatonin disappeared in the presence of a CREB inhibitor (Figure 4H). To verify the interaction between CREB and the *ATG3* promoter, we constructed vectors containing the *ATG3* promoter sequence with the CREB binding site (Figure 4I). We then performed a luciferase assay to confirm the interaction of CREB and *ATG3* promoter, and the results showed that the luciferase activity in the group co‐transfected with plasmids carrying CREB‐CDS and *ATG3* promoter sequences was notably increased, compared to that in the group co‐transfected with vectors carrying CREB‐CDS and a blank vector without the *ATG3* promoter sequence (Figure 4J). Taken together, these findings indicated that after melatonin binds to its receptors MT1/2, the transcription factor CREB is initiated and it further upregulates the expression of *ATG3*, which is responsible for melatonin‐repressed apoptosis in HCAECs, in an autophagy‐dependent manner.

### 3.5 *Atg3* deficiency blocks the protective effects of melatonin in the KD mouse model

**FIGURE 5:**
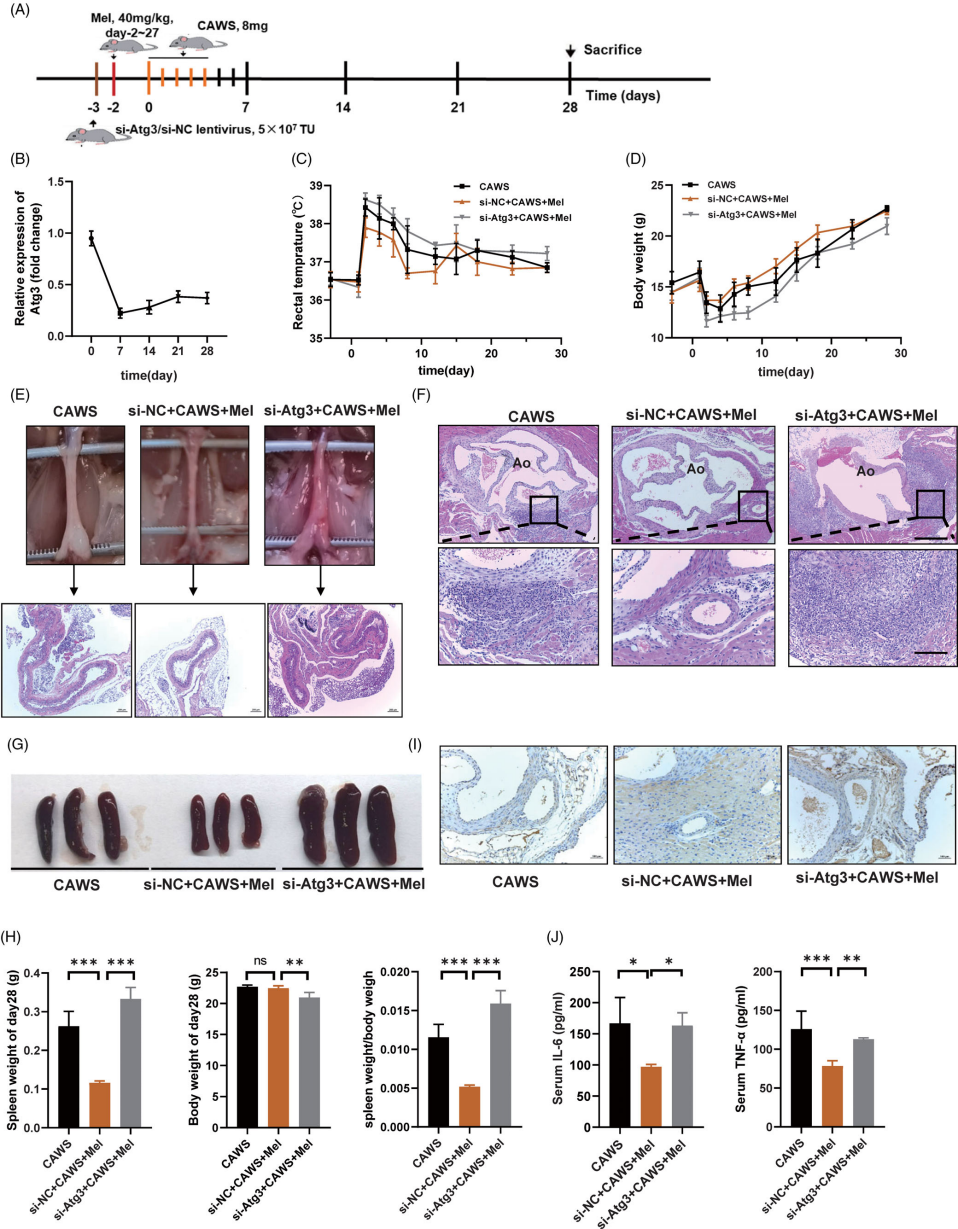
ATG3 deficiency blocks the protective effects of melatonin in the KD mouse model. (A) Modelling timeline for the CAWS (CAWS, 8 mg), si‐NC+CAWS+melatonin (melatonin, 40 mg/kg), and si‐*Atg3*+CAWS+melatonin groups. (B) Relative expression of *Atg3* in the heart tissues of mice after *Atg3*‐knockdown, as analysed using RT‐qPCR. (C) Daily change of rectal temperature of mice in the CAWS, si‐NC+CAWS+melatonin, and si‐*Atg3*+CAWS+melatonin groups. (D) Daily change of body weight of mice in the CAWS, si‐NC+CAWS+melatonin, and si‐*Atg3*+CAWS+melatonin groups. (E) H&E staining of the abdominal aorta. Scale bar: 200 μm. (F) H&E staining of the aortic root. The scale bar of the upper pictures is 200 μm, while that of the bottom pictures is 50 μm. (G) Representative images of spleens of mice on day 28. (H) Spleen weights, body weights, and the ratio of the two on day 28. (I) Immunohistochemistry showing the expression of Bcl‐2 in the coronary arteries of mice. (J) Serum concentrations of TNF‐α and IL‐6 in the mice on day 14, as detected using ELISA. Data are presented as mean ± SEM. *p < 0.05, **p < 0.01, and ***p < 0.001; ns, not significant. *Atg3*, autophagy‐related gene‐3; CAWS, *Candida albicans* water‐soluble fraction; KD, Kawasaki disease; NC, negative control

Next, to determine whether *Atg3* is responsible for the protective effect of melatonin in CAWS‐induced KD vasculitis, we injected a lentivirus carrying shRNA into mice, to silence *Atg3*, and then administered melatonin according to the modelling timeline (Figure 5A). Upon injecting shRNA, there was a remarkable reduction in the *Atg3* expression in the heart tissues of KD mice (Figure 5B). Mice showed more severe fever and weight loss in the absence of *Atg3* (Figure 5C,D). The previously remitted abdominal aortitis reappeared after silencing of *Atg3* (Figure 5E). H&E staining showed that the severe inflammatory cell infiltration around the aortic root and coronary artery that was previously improved by melatonin treatment was observed again after silencing of *Atg3* (Figure 5F). Recurrence of splenomegaly was also observed after silencing of *Atg3* (Figure 5G,H). Immunohistochemistry revealed that there was a decrease in the expression of Bcl‐2 in the cardiovascular tissues of KD mice in the *Atg3*‐silenced group (Figure 5I). ELISA showed that serum levels of TNF‐α and IL‐6 were elevated after silencing of *Atg3* in KD mice (Figure 5J). Taken together, these findings indicated that silencing of *Atg3* blocked the protective effects of melatonin in KD mice in vivo.

### 3.6 Melatonin inhibits macrophage‐mediated immunopathological damage of vascular endothelial cells in patients with KD

**FIGURE 6:**
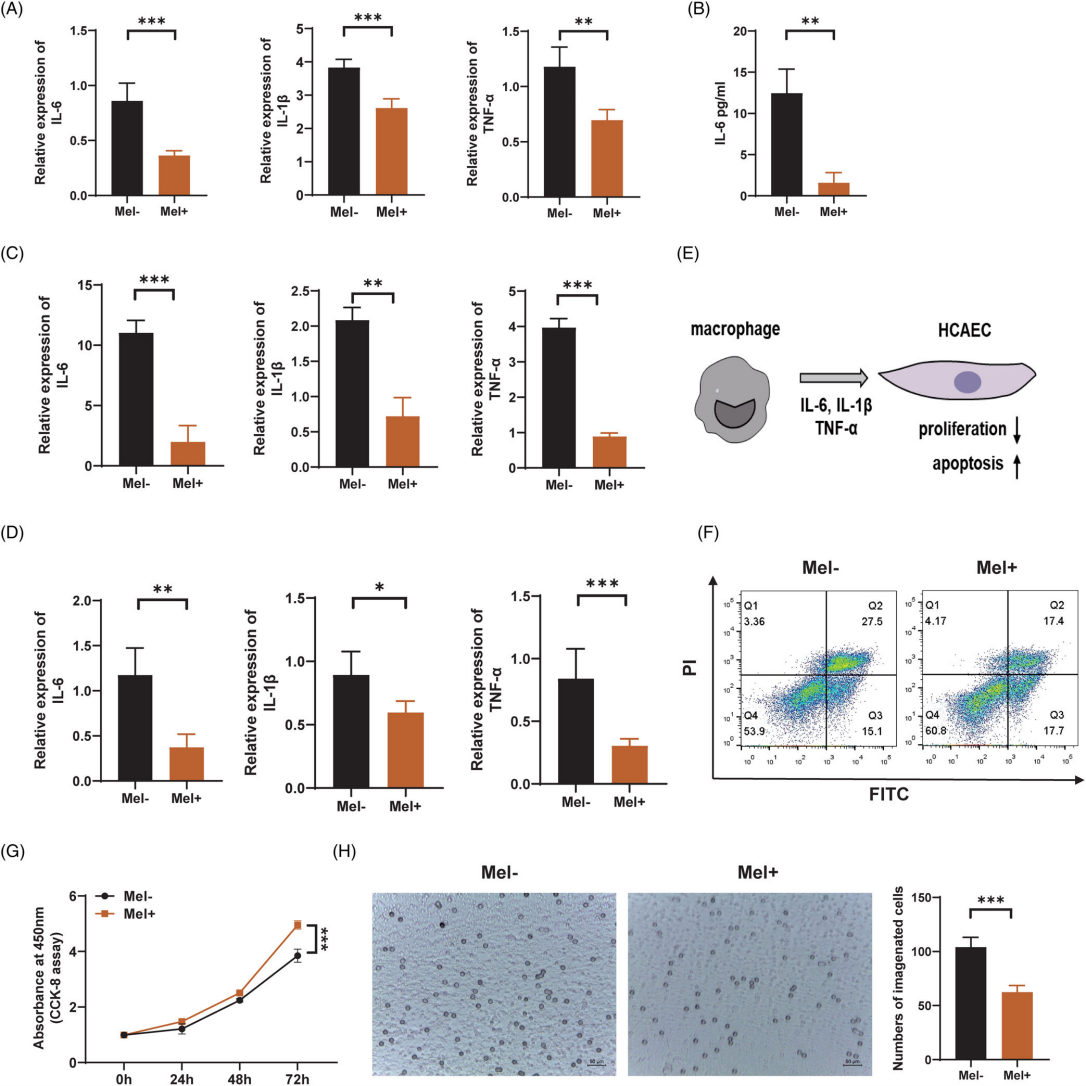
Melatonin inhibits macrophage‐mediated immunopathological damage of vascular endothelial cells in patients with Kawasaki disease. (A) Relative expression of *IL‐6*, *IL‐1β*, and *TNF‐α* in LPS (100 ng/ml)‐stimulated THP1 cells, with or without melatonin (0.5 mM) pre‐treatment, as detected using RT‐qPCR. (B) ELISA analysis showing the protein level of IL‐6 in the cell culture supernatant of THP1 cells treated with LPS (100 ng/ml) and melatonin (0.5 mM). (C) Relative expression of *IL‐6*, *IL‐1β*, and *TNF‐α* in TNF‐α (20 ng/ml)‐stimulated macrophages, as detected using RT‐qPCR. (D) Relative expression of *IL‐6*, *IL‐1β*, and *TNF‐α* in PBMC‐derived macrophages of KD patients, with or without melatonin (0.5 mM) pre‐treatment. (E) An in vitro co‐culture system of HCAECs and macrophages. (F) Apoptosis of HCAECs co‐cultured with macrophages, as assessed using Annexin V‐FITC/PI double‐staining and flow cytometry. (G) Cell proliferation of HCAECs co‐cultured with macrophages, as assessed using CCK8 assay. (H) Microscopic images showing the number of THP1‐derived macrophages migrating to TNF‐α‐stimulated HCAECs, as detected using transwell assay. Scale bar: 50 μm. Data are presented as mean ± SEM. *p < 0.05, **p < 0.01, ***p < 0.001; ns, not significant. IL‐1β, interleukin‐1 beta; IL‐6, interleukin‐6; LPS, lipopolysaccharide; PBMC, peripheral blood mononuclear cells; TNF‐α, tumour necrosis factor alpha

Given that KD‐related vasculitis is a crosstalk between vascular endothelial cells and immune cells, we questioned whether melatonin may also inhibit inflammatory responses mediated by immune cells in KD‐related vasculitis. Previous studies have demonstrated that during the pathogenesis of KD, macrophages can release inflammatory cytokines to mediate damage to vascular endothelial cells.^32^, ^34^, ^35^ A recent single cell sequencing study revealed that monocytes are the main source of differentially expressed genes, and pro‐inflammatory genes such as *IL‐1β* and *TNF‐α* are significantly upregulated in PBMCs of KD patients, suggesting a pivotal role of monocytes/macrophages in KD vasculitis.^36^ Therefore, we investigated the effects of melatonin on macrophage‐mediated inflammation. Subsequent RT‐qPCR results demonstrated remarkably decreased mRNA levels of *IL‐6*, *IL‐1β*, and *TNF‐α*, in LPS‐stimulated human THP1‐derived macrophages pre‐treated with melatonin (Figure 6A). Consistently, ELISA analysis showed a decreased protein level of IL‐6 in the culture supernatant of melatonin‐treated THP1 cells (Figure 6B). TNF‐α is the one of the leading cytokines mediating KD vasculitis, in order to better mimic the activation of macrophages in KD, we also investigated the anti‐inflammatory role of melatonin in TNF‐α‐stimulated human THP1‐derived macrophages. Consistent with previous results, subsequent RT‐qPCR results demonstrated remarkably decreased mRNA levels of *IL‐6*, *IL‐1β*, and *TNF‐α* (Figure 6C). To consolidate our findings, we separated PBMCs from KD patients and induced them into macrophages using rhM‐CSF. RT‐qPCR results showed that the mRNA levels of *TNF‐α*, *IL‐1β*, and *IL‐6* were lower in the melatonin‐treated human PBMC‐derived macrophages than in the PBS‐treated human macrophages, which was consistent with the results observed in THP1‐derived macrophages (Figure 6D). Next, to investigate whether melatonin alleviated the HCAEC damage by reducing the levels of pro‐inflammatory cytokines secreted by macrophages, we established an in vitro co‐culture system (Figure 6E). Intriguingly, HCAECs cultured with the supernatant of LPS‐stimulated THP1‐derived macrophages pre‐treated with melatonin exhibited repressed apoptosis (Figure 6F) and enhanced proliferation (Figure 6G). To further investigate the role of melatonin in the interaction between macrophages and vascular endothelial cells, we explored the effect of melatonin on the chemotaxis of HCAECs to macrophages, transwell assay showed that there was a significant decrease in the number of macrophages migrating towards HCAECs in the group pre‐treated with melatonin (Figure 6H). These data highlight the critical role of melatonin in regulating the crosstalk between HCAECs and macrophages in KD‐related vasculitis.

## 4 DISCUSSION

**FIGURE 7:**
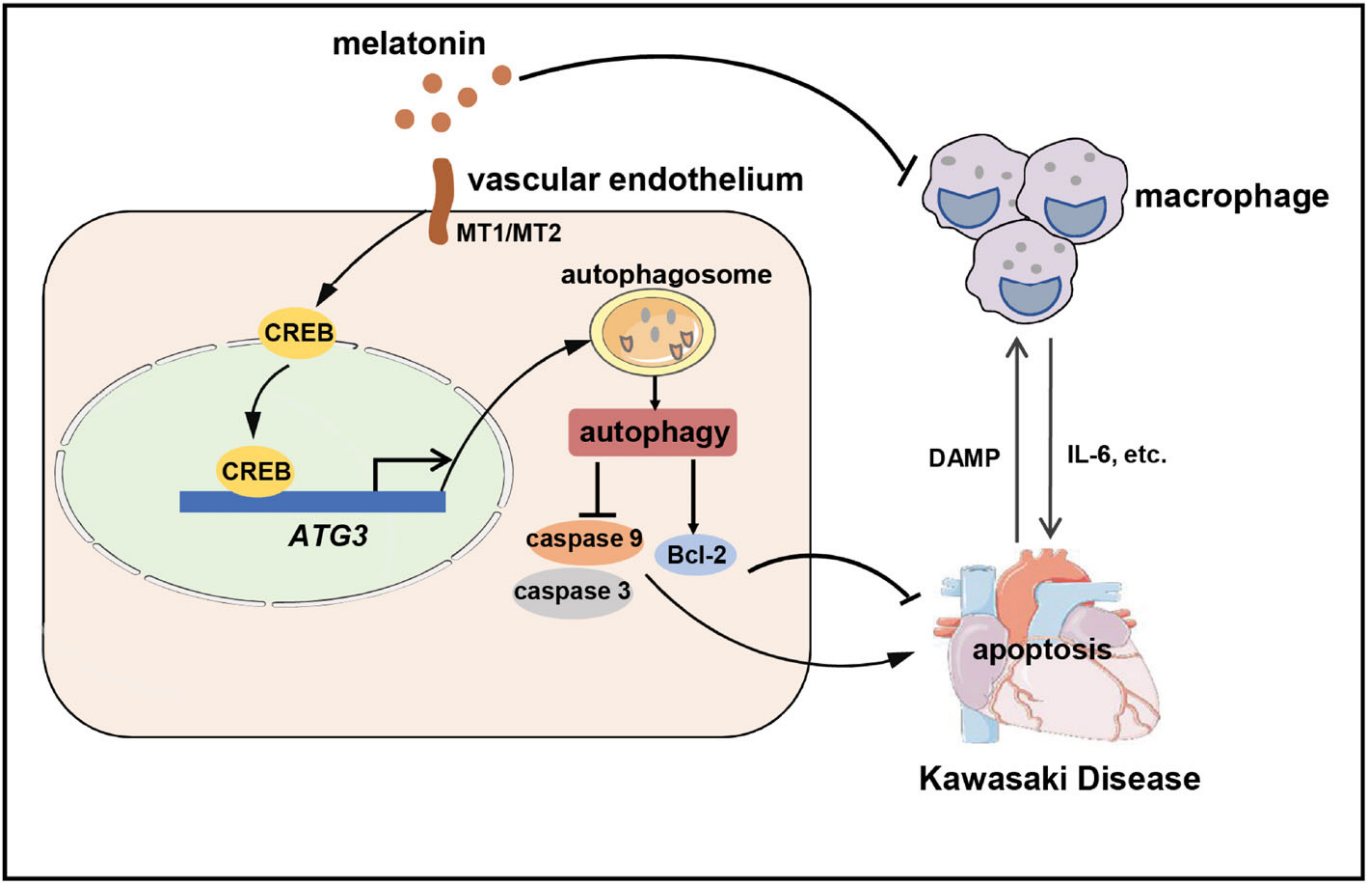
A schematic model of the function of melatonin in KD‐related vasculitis. KD‐related vasculitis is a crosstalk between vascular endothelial cells and immune cells, especially macrophages. Macrophages release pro‐inflammatory cytokines (such as IL‐6) to mediate damage to the vascular endothelial cells, and the damaged vascular endothelial cells in turn release more damage‐associated molecular patterns (DAMPs), to promote the secretion of pro‐inflammatory cytokines from the macrophages. Melatonin alleviates vascular endothelial cell injury directly, by suppressing apoptosis in an autophagy‐dependent manner, and also decreases the production of the pro‐inflammatory cytokines released by macrophages, thereby reducing the immunopathological damage of vascular endothelial cells in KD‐related vasculitis. *ATG3*, autophagy‐related gene‐3; CREB, cAMP response element‐binding protein; DAMP, damage‐associated molecular pattern; KD, Kawasaki disease; MT1, melatonin receptor 1; MT2, melatonin receptor 2

In the present study, we found that melatonin alleviated KD‐related vascular endothelial cell damage both in vitro and in vivo, by activating the melatonin/MT/CREB pathway and subsequent *ATG3* expression, thereby suppressing vascular endothelial cell apoptosis in an autophagy‐dependent manner. Additionally, melatonin reduced the production of pro‐inflammatory cytokines released by macrophages and indirectly reduced the immunopathological damage to vascular endothelial cells (Figure 7). Taken together, our findings revealed the potential of melatonin as a therapeutic alternative for KD.

CREB is a transcription factor that plays a crucial role in regulating cell proliferation, apoptosis, and autophagy, by inducing the expression of related genes. Melatonin exerts its protective effects by regulating CREB in a broad spectrum of diseases. Melatonin overcomes the disrupted brain energy homeostasis and boosts neuroinflammation, thereby protecting against brain damage, by regulating the phospho‐5′AMP‐activated protein kinase/CREB signalling pathways in traumatic brain injury.^37^ Another study revealed that melatonin improved cognitive and mood impairments by modulating oxidative stress, NLRP3 inflammasomes, and the BDNF (brain‐derived neurotrophic factor)/ERK (extracellular signal‐regulated kinase)/CREB pathway in a rat model of chronic Gulf War Illness.^38^ The interaction between CREB and autophagy also plays a pivotal role in the occurrence and development of diseases.^39^ CREB mediates the expression of autophagy‐related genes.^40^ Our present study identified a crucial role of CREB in the maintenance of the autophagy‐promoting effect of melatonin. Melatonin binds to MT1/2 receptors and activates CREB, to upregulate the target gene *ATG3*, thereby exerting an anti‐apoptotic effect in HCAECs, in an autophagy‐dependent manner. These findings provide a novel mechanism for the autophagy‐modulating effects of melatonin in KD.

Splenomegaly was an obvious feature observed in CAWS‐induced murine vasculitis in the present study, but it has not been described in detail in previous reports. Splenomegaly is observed in clinical KD patients and is associated with hyper‐activated inflammatory responses manifested by excessive production of a wide array of inflammatory cytokines in KD‐macrophage activation syndrome (MAS).^41^ Considering the critical role of splenomegaly in the vascular pathology of KD, we believe that the CAWS‐induced murine vasculitis model could simulate KD‐related vascular pathology.

MAS is a hyper‐activated inflammatory state characterized by the activation and expansion of T lymphocytes and hemophagocytic macrophages that eventually leads to a series of clinical manifestations, including persistent fever, cytopenia, liver dysfunction, coagulopathy, and extreme hyperferritinemia.^42^, ^43^, ^44^ Han et al. showed that the existence of MAS should be considered when a KD patient shows persistent fever, splenomegaly, cytopenia, hyperferritinemia, or IVIG resistance.^35^ Therefore, relief of the hyper‐inflammatory state of MAS may contribute substantially to the reduced burden of KD, and melatonin could be used as an agent for that purpose, since it regulates macrophage‐mediated inflammation.^45^, ^46^ In the present study, we observed that melatonin significantly suppressed the levels of pro‐inflammatory cytokines such as IL‐1β and IL‐6 in THP1‐derived macrophages, as well as, human PBMC‐derived macrophages in KD patients. In addition, we demonstrated that melatonin alleviated vascular endothelial cell damage partly by modulating macrophage activation, suggesting that macrophages may be a therapeutic target for KD‐related vascular damage.

In summary, our study provides theoretical foundation for the use of melatonin as an additional therapy to further reduce the incidence of CAA in KD, and presents a new concept that an imbalance in homeostasis of the autophagy‐apoptosis axis might be the underlying pathological mechanism of KD. In addition, *ATG3* and autophagy are the other possible therapeutic targets for KD, and by virtue of several biological or physical materials, *ATG3*‐ or autophagy‐based treatments may be achieved in future. Our study also provides evidence that suppressing macrophage activation is a strategy for the treatment of KD. The activation of macrophages is directly related to the occurrence, development, and prognosis of a variety of inflammatory diseases, such as juvenile idiopathic arthritis, inflammatory bowel disease, and atherosclerosis. Therefore, our findings would be of great interest to a broader scientific community.

### 4.1 Limitations of this study

One limitation of this study is that we recruited only 16 patient samples, and this small sample size limited the accuracy of assessing the anti‐inflammatory effect of melatonin in PBMC‐derived macrophages from KD patients. Another limitation of the study is that it does not explore the detailed mechanism of the anti‐inflammatory effect of melatonin in macrophages.

## AUTHOR CONTRIBUTIONS

Yuanzheng Zheng, Xiao Han, and Yonghao Gui contributed to the conception and design of the study. Yuanzheng Zheng and Saihua Huang contributed to data collection. Jialing Zhang, Jia Hou, Fang Wu, and Wenji Wang performed the statistical analyses. Yuanzheng Zheng wrote the first draft of the manuscript. Saihua Huang, Xiao Han, and Jialing Zhang wrote sections of the manuscript. All authors contributed to the manuscript revision and approved the submitted version.

## CONFLICT OF INTEREST

The authors declare that they have no conflicts of interest.

## Supporting information


**TABLE S1** Clinical characteristics of KD patients
**TABLE S2** Treatments and representative laboratory data of KD patients
**TABLE S3** Levels of urinary 6‐sulfatoxymelatonin of recruited donors
**TABLE S4** Sequences of si‐RNAs targeting human *ATG3* gene (5′‐3′)
**TABLE S5** The sequences of primers for qRT‐PCR (5′‐3′)
**TABLE S6** Sequences of primers for the amplification of *CREB* full length and *ATG3* promoter (5′‐3′)
**TABLE S7** Sequences of shRNAs carried by lentivirus vector (5′‐3′)
**FIGURE S1** Flow chart of KD patient selection process
**FIGURE S2** Kinetics of autophagic flux was detected by fluorescence microscope after HCAECs was infected with adenoviral vector expressing mRFP‐GFP‐LC3. ***p < 0.001. Mel, melatonin group (melatonin, 0.5 mM, 6 h); si‐ATG3+Mel, melatonin treatment after silencing of *ATG3*; NC, negative control

## Data Availability

The data referred to in this study have been deposited in Gene Expression Omnibus database, with the accession number GSE183359.
