# Supplementary material for: Melatonin alleviates vascular endothelial cell damage by regulating an autophagy‐apoptosis axis in Kawasaki disease
Source: Cell Prolif. 2022 May 17;55(6):e13251. doi: 10.1111/cpr.13251 (PMC9201377; doi:10.1111/cpr.13251)
Supplement: Supplementary file 1 — TABLE S1 Clinical characteristics of KD patients TABLE S2 Treatments and representative laboratory data of KD patients TABLE S3 Levels of urinary 6‐sulfatoxymelatonin of recruited donors TABLE S4 Sequences of si‐RNAs targeting human ATG3 gene (5′‐3′) TABLE S5 The sequences of primers for qRT‐PCR (5′‐3′) TABLE S6 Sequences of primers for the amplification of CREB full length and ATG3 promoter (5′‐3′) TABLE S7 Sequences of shRNAs carried by lentivirus vector (5′‐3′) FIGURE S1 Flow chart of KD patient selection process FIGURE S2 Kinetics of autophagic flux was detected by fluorescence microscope after HCAECs was infected with adenoviral vector expressing mRFP‐GFP‐LC3. ***p < 0.001. Mel, melatonin group (melatonin, 0.5 mM, 6 h); si‐ATG3+Mel, melatonin treatment after silencing of ATG3; NC, negative control [file CPR-55-e13251-s001.docx]

**Supplementary Table 1. Clinical characteristics of KD patients**

| Patients | 1 | | 2 | 3 | 4 | 5 | 6 | 7 | 8 | 9 | 10 | 11 | 12 | 13 | 14 | 15 | 16 |
| --- | --- | --- | --- | --- | --- | --- | --- | --- | --- | --- | --- | --- | --- | --- | --- | --- | --- |
| Age(years) | | 1.4 | 3.9 | 1.6 | 5.1 | 3.6 | 1.8 | 2.5 | 1.3 | 2.7 | 3.2 | 1.7 | 2 | 2.5 | 4.9 | 3.7 | 5 |
| Gender | | M | M | M | F | M | F | M | M | F | F | M | M | F | M | M | F |
| Fever(days) | | 6 | 6 | 7 | 9 | 6 | 7 | 7 | 7 | 8 | 7 | 6 | 7 | 7 | 6 | 6 | 8 |
| Conjunctivitis(yes/no) | | Y | Y | Y | Y | N | Y | Y | Y | Y | Y | Y | Y | Y | Y | Y | Y |
| Oral changes(yes/no) | | Y | Y | Y | Y | Y | Y | Y | Y | Y | N | Y | Y | Y | Y | Y | Y |
| Extremity changes(yes/no) | | Y | N | Y | Y | Y | N | N | Y | Y | Y | N | N | Y | Y | Y | Y |
| Rash(yes/no) | | Y | Y | Y | Y | Y | Y | Y | Y | Y | Y | Y | Y | Y | Y | N | Y |
| Cervical lymphadenopathy(yes/no) | | Y | Y | Y | Y | Y | Y | Y | Y | Y | Y | Y | Y | Y | Y | Y | Y |
| Complete KD (yes/no) | | Y | Y | Y | Y | Y | Y | Y | Y | Y | Y | Y | Y | Y | Y | Y | Y |
| CAL (yes/no) | | N | N | N | N | N | N | N | N | N | N | N | Y | N | N | N | N |

Abbreviation: CAL, coronary artery lesion.

**Supplementary Table 2. Treatments and representative laboratory data of KD patients**

| Patients | 1 | 2 | 3 | 4 | 5 | 6 | 7 | 8 | 9 | 10 | 11 | 12 | 13 | 14 | 15 | 16 |
| --- | --- | --- | --- | --- | --- | --- | --- | --- | --- | --- | --- | --- | --- | --- | --- | --- |
| Sample collection time (days after onset) | 6 | 6 | 7 | 9 | 6 | 7 | 7 | 7 | 8 | 7 | 6 | 7 | 7 | 6 | 6 | 8 |
| IVIG (yes/no) | Y | Y | Y | Y | Y | Y | Y | Y | Y | Y | Y | Y | Y | Y | Y | Y |
| Aspirin (yes/no) | Y | Y | Y | Y | Y | Y | Y | Y | Y | Y | Y | Y | Y | Y | Y | Y |
| Steroids (yes/no) | N | N | N | N | N | Y | N | N | N | N | N | N | N | N | N | Y |
| Other treatments (yes/no) | N | N | N | N | N | N | N | N | N | N | N | N | N | N | N | N |
| IVIG resistance (yes/no) | N | N | N | N | N | Y | N | N | N | N | N | N | N | N | N | Y |
| WBC(X10*9/L) | 24.3 | 20.2 | 5.7 | 11.0 | 18.4 | 10.6 | 14.2 | 15.9 | 16.4 | 14.5 | 13.1 | 17.9 | 8.0 | 10.5 | 14.8 | 17.9 |
| RBC(X10*12/L) | 4.06 | 4.20 | 4.54 | 4.44 | 4.16 | 3.86 | 4.84 | 4.37 | 4.33 | 3.89 | 3.55 | 4.21 | 4.47 | 4.42 | 3.80 | 4.61 |
| Hb (g/L) | 108 | 112 | 116 | 125 | 118 | 111 | 123 | 124 | 119 | 106 | 95 | 111 | 109 | 121 | 108 | 124 |
| PLT(X10*9/L) | 454 | 293 | 230 | 399 | 290 | 213 | 209 | 523 | 218 | 412 | 426 | 541 | 233 | 458 | 438 | 262 |
| ESR (mm/h) | 106 | 108 | 78 | 91 | 87 | 84 | 115 | 39 | 85 | 33 | 120 | 118 | 88 | 96 | 39 | 119 |
| CRP (mg/L) | 53 | 100 | 12 | 63 | 61 | 89 | 44 | 21 | 160 | 76 | 102 | 73 | 64 | 44 | 63 | 162 |

KD, Kawasaki disease; IVIG, intravenous immunoglobulins; WBC, white blood cell; RBC, red blood cell; Hb, hemoglobin; PLT, platelet; ESR, erythrocyte sedimentation rate; CRP, C-reactive protein

**Supplementary Table 3. Levels of urinary 6-sulfatoxymelatonin of recruited donors**

| Patients | urinary 6-sulfatoxymelatonin concentration (ng/mL) |
| --- | --- |
| P1 | 30.602 |
| P2 | 13.487 |
| P3 | 22.450 |
| P4 | 17.537 |
| P5 | 26.025 |
| P6 | 21.947 |
| P7 | 51.323 |
| P8 | 36.445 |
| P9 | 26.204 |
| P10 | 37.260 |
| P11 | 18.395 |
| P12 | 10.897 |
| P13 | 44.380 |
| P14 | 12.307 |
| P15 | 28.141 |
| P16 | 12.307 |

**Supplementary Table 4. Sequences of si-RNAs** **targeting human *ATG3* gene (5’-3’).**

| siRNA | Sequences (5’-3’) |
| --- | --- |
| *ATG3*-Homo-841 | F: GGGAGAACUUGGAGUUCAUTT |
|  | R: AUGAACUCCAAGUUCUCCCTT |
| *ATG3*-Homo-458 | F: GCUGCAGAUAUGGAAGAAUTT |
|  | R: AUUCUUCCAUAUCUGCAGCTT |
| Negative control | F: UUCUCCGAACGUGUCACGUTT |
|  | R: ACGUGACACGUUCGGAGAATT |

**Supplementary Table 5. The sequences of primers for qRT-PCR (5’-3’).**

| Genes | Sequences (5’-3’) |
| --- | --- |
| Human *MMP-9* | F: GCCACTACTGTGCCTTTGAGTC |
|  | R: CCCTCAGAGAATCGCCAGTACT |
| Human *E-selectin* | F: TCAAGGGCAGTGGACACAGCAA |
|  | R: GGAAACTGCCAGAAGCACTAGG |
| Human *VCAM-1* | F: GATTCTGTGCCCACAGTAAGGC |
|  | R: TGGTCACAGAGCCACCTTCTTG |
| Human *ICAM-1* | F: AGCGGCTGACGTGTGCAGTAAT |
|  | R: TCTGAGACCTCTGGCTTCGTCA |
| Human *Bcl-2* | F: ATCGCCCTGTGGATGACTGAGT |
|  | R: GCCAGGAGAAATCAAACAGAGGC |
| Human *FLIP* | F: AGTGAGGCGATTTGACCTGCTC |
|  | R: CCTCACCAATCTCTGCCATCAG |
| Human *ATG3* | F: ACTGATGCTGGCGGTGAAGATG |
|  | R: GTGCTCAACTGTTAAAGGCTGCC |
| Human *IL-6* | F: AGACAGCCACTCACCTCTTCAG |
|  | R: TTCTGCCAGTGCCTCTTTGCTG |
| Human *IL-1β* | F: CCACAGACCTTCCAGGAGAATG |
|  | R: GTGCAGTTCAGTGATCGTACAGG |
| Human *TNF-α* | F: CTCTTCTGCCTGCTGCACTTTG |
|  | R: ATGGGCTACAGGCTTGTCACTC |
| Human *ACTB* | F: CACCATTGGCAATGAGCGGTTC |
|  | R: AGGTCTTTGCGGATGTCCACGT |
| Mouse *Atg3* | F: TAAGGCTGACGCTGGAGGTGAA |
|  | R: GTGCTCAACTGTTAAAGGCTGCC |
| Mouse *Actb* | F: CATTGCTGACAGGATGCAGAAGG |
|  | R: TGCTGGAAGGTGGACAGTGAGG |

**Supplementary Table 6. Sequences of primers for the amplification of *CREB* full length and *ATG3* promoter (5’-3’).**

| Genes | Sequences (5’-3’) |
| --- | --- |
| CREB-F-*Hind* III | CAAGCTTATGACCATGGAATCTGGAGCCGAG |
| CREB-R-*Xba* I | CCTCTAGAATCTGATTTGTGGCAGTAAAGGTCC |
| ATG3 promoter-F-*Nhe* I | GGCTAGCGTTGATACAATATGACCGCACAATAT |
| ATG3 promoter-R-*Xho* I | CCTCGAGTAGCAGAACAGGAAAAGGTGAGAGA |

**Supplementary Table 7. Sequences of shRNAs carried by lentivirus vector (5’-3’).**

| shRNA | Sequences (5’-3’) |
| --- | --- |
| LV3-*Atg3*-Mus-553 | GGAAGCTATCATTGAAGAAGA |
| LV3-*Atg3*-Mus-591 | GGGTAGATACATATCACAACA |
| Negative control | TTCTCCGAACGTGTCACGT |

**Supplementary figure 1**


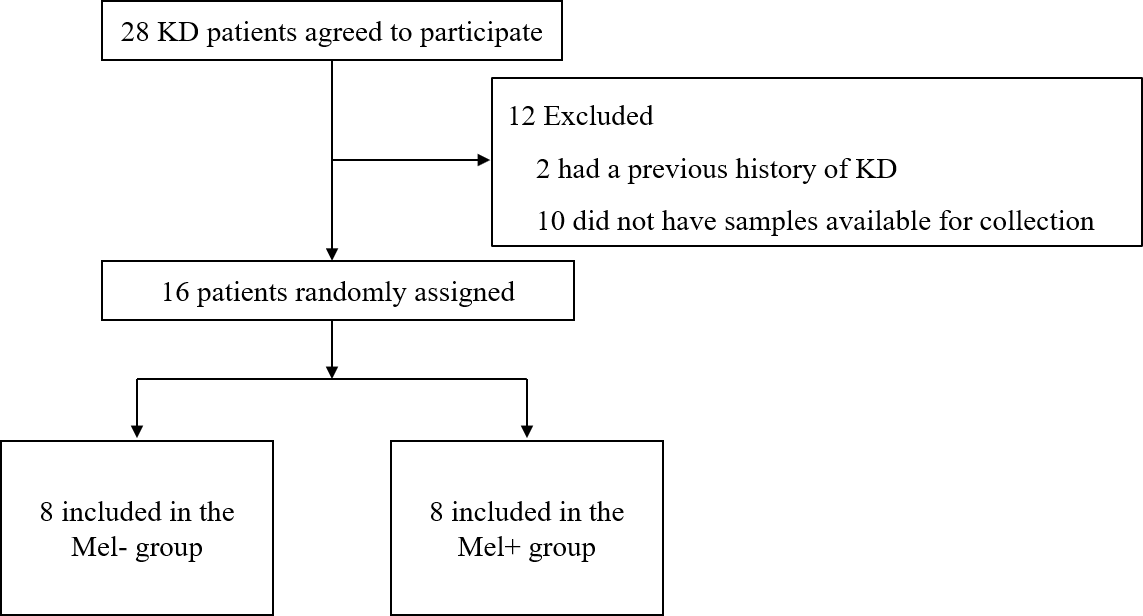


**Supplementary figure 1.** Flow chart of KD patient selection process.

**Supplementary figure 2**

**Supplementary figure 2**. Kinetics of autophagic flux was detected by fluorescence microscope after HCAECs was infected with adenoviral vector expressing mRFP-GFP-LC3. ***P<0.001. Mel, melatonin group (melatonin, 0.5 mM, 6 h); si-ATG3+Mel, melatonin treatment after silencing of *ATG3*; NC, negative control
